# Early exposure to sugar sweetened beverages or fruit juice differentially influences adult adiposity

**DOI:** 10.1038/s41430-024-01430-y

**Published:** 2024-03-15

**Authors:** David Benton, Hayley A. Young

**Affiliations:** School of Psychology, Swansea University, Swansea, SA2 8PP United Kingdom

**Keywords:** Nutrition, Ageing

## Abstract

**Objective:**

To examine associations between different types of sweet drinks consumed in early life and adult adiposity.

**Design:**

The analysis involved the secondary analysis of the Avon Longitudinal Study of Parents and Children which followed children from birth to 24 years. Adiposity was measured using Dual-energy X-ray absorptiometry while food frequency questionnaires and diaries monitored diet. ‘Early exposure’ to sweet drinks was defined as giving a sugar-sweetened beverage or 100% fruit juice (FJ), before two years of age.

**Results:**

Early exposure to cola was associated with higher fat mass, android fat mass and BMI at age 24 years; whereas early exposure to apple juice was associated with lower adult adiposity in females but not males. When age three, exposure to cola was associated with a greater intake of energy, carbohydrates, protein, fat, and less fruit and more fried foods. In contrast, early exposure to apple juice was associated with higher protein and lower fat intakes and consuming more fruits/vegetables and less fried foods. Parental education, adiposity and socio-economic status influenced whether SSB or FJ was given to a child.

**Conclusions:**

Early drinking of sugar sweetened beverages was associated with a less healthy dietary pattern, and greater adult adiposity. Early drinking of apple juice was associated with a healthier dietary pattern, and lower fat mass in adult females. The choice of drink was associated with social deprivation. As the dietary causes of adult obesity begin in early childhood, increased attention should be given to diet in the first years of life.

## Introduction

There are many reports that consuming sugar sweetened drinks (SSB) in childhood is associated with a greater risk of obesity, and some studies have treated all sweet drinks as similar; for example combining carbonated drinks, sweetened tea, energy drinks, fruit-based drinks, and 100% fruit juice with no added sugar (FJ) [1]. Others have attempted to differentiate between SSB and drinks such as FJ that contain endogenous sugar, often reporting a lack of association between FJ and body weight [2–5]. There are, however, reviews that come to the opposite conclusion. Based on eight prospective cohorts, Hebden [6] found that drinking fruit juice increased body weight, although seven of the eight studies involved simply asking how frequently ‘fruit juices’ were consumed, without distinguishing 100% juices from those to which sugar had been added. The question therefore considered, is whether we should differentiate between SSB, FJ, and juice-containing drinks with added sugar, when considering how beverages in childhood influence longer-term weight.

It is, however, acknowledged that those consuming one food, will be more likely to choose other foods, forming a dietary pattern [7–9]. The Standard American or Western pattern diet is characterized by a high intake of refined and processed foods, that is high in both fat and sugar. Importantly those who eat this diet are more likely to choose SSB [10]. As such, SSB may be a marker for the choice of a highly calorific diet, so that changing what is drunk will have a limited impact on energy intake.

The present interest in the diet during childhood reflects that early diet is a risk factor for adult obesity, and the influence of SSB has been less studied in the young. Are SSB gateway foods that encourage the consumption of sweetened foods in later life, or a marker for a later obesity-inducing dietary pattern? If so, should attention be directed to a dietary pattern, rather than a single nutrient or source of that nutrient?

The study therefore considered whether all sugar drinks should be treated as a single category, or alternatively whether the influence of each should be examined separately. Secondly it was considered whether the impact of a sweet drink should be viewed as part of a dietary pattern. Thirdly whether males and females respond differently when the choice of drink was examined.

## Methods

### The Avon Longitudinal Study of Parents and Children study (ALSPAC)

The sample comprised women recruited in the Bristol area of the United Kingdom, who gave birth between 1st April 1991 and 31st December 1992 [11, 12] and included 14,541 pregnancies that resulted in 13,988 children alive at one year.

### Height, weight and adiposity

Height was measured to the nearest 0.1 cm using a stadiometer (Holtain Ltd, Crymych Pembrokeshire, UK), and weight to the nearest 0.1 kg using Tanita scales (Wardworth Ltd, Bolton, UK). Halfway between the lower ribs and the pelvic bone, a tape was used to measure waist circumference to the nearest 1 mm. Body mass index (BMI) was calculated by dividing the weight in kilograms by the height in metres squared.

### Dual-energy X-ray absorptiometry (DEXA scan)

A Lunar Prodigy narrow fan-beam densitometer (GE Healthcare, Bedford, UK) was used for whole-body scanning. Android fat mass measures abdominal fat around the organs and in this respect differs from subcutaneous fat. Total fat mass at 24 years was used as the dependent variable.

### Diet

When the children were two years of age, the carer was asked if between 15 months and 2 years the child drank cola, other fizzy drinks, apple juice, other fruit juices or fruit squash (a drink containing some fruit juice to which sugar was added), eliciting a yes/no response, although the amount consumed was not recorded. For the purposes of the present analysis, ‘early exposure’ was defined as any consumption of the above drinks before 2 years of age.

The child’s diet at age 3 years was assessed using a Food Frequency Questionnaire completed by the mother or partner. From the foods available, to gain a general impression of the dietary style, various foods were examined as falling into the following groups. The choice of food items was based on an analysis of dietary patterns in this cohort that establish a processed food and healthy pattern of consumption [13].

1. Fatty foods: Burger and sausages; French fries; Fried food.
2. Sweet tasting foods: Chocolate; Sweets; Biscuits; Cakes; Puddings.
3. Fruit and vegetables: Fresh fruit; Green vegetables; Root vegetables; Salad.
4. Other foods; Fish; Meat; Pizza.

Each food item was rated as having been eaten ‘never/rarely,’ ‘once in 2 weeks,’ ‘1–3 times/week,’ ‘4–7 times/week,’ or ‘more than once a day.’ These responses were modified to reflect weekly consumption: never/rarely was given a score of zero; once in two weeks scored 0.5; 1–3 a week scored 2; 4–7 a week scored 5.5; more than once a day scored 10.

Subsequently parents completed a three-day dietary diary (one weekend day and two weekdays), when the child was 4 and 7 years of age. At 11 and 13 years of age the child kept a similar record. The records were checked with the parents or children, and were used to calculate the daily mean intakes [14] using the McCance and Widdowson’s Food Tables [15]. Free sugars reflected any monosaccharides or disaccharides that had been added to foods, plus sugars occurring naturally in honey, syrups, and fruit juices. Misreporting of dietary data was assessed at age 11 and 13-years using estimates of energy requirement based on age, sex and body weight, and the ratio between the reported intake and estimated need [16].

### Demographics and confounding variables

#### Partner’s education

The highest qualification achieved was classified as Certificate of Secondary Education/Vocational Education; O-levels; A-levels; Degree (higher scores were given for longer education).

#### Mother’s and partner’s occupational status

Social class was classified as 1) Professional jobs, 2) Managerial and technical, 3) Skilled non-manual, 4) Skilled manual, 5) Partly skilled, 6) Unskilled (lower scores given for higher status).

#### Index of multiple deprivation

Various domains were combined to create a single dimension incorporating income; employment; health deprivation and disability; education, skills and training; housing [17].

Data were also collected on partner’s BMI, age of mother at the child’s birth, and the mother’s prenatal weight.

### Statistical analysis

Data were collected and managed using REDCap electronic data capture tools (Research Electronic Data Capture) [18]. Regression equations were produced using the General Linear Model, with drinks added as dummy variables to examine the effect of drink on adiposity (SPSS version 28.0.1, IBM Corporation, Armonk, New York). However, as males differ in adiposity from females, and the response to the drinks differed, each gender was studied separately. Hierarchical regression equations were used to consider the influence of factors other than drink, with the type of drink added to create a second model. Collinearity was examined, using the variance inflation factor, to remove variables that were highly correlated. On all occasions two-sided tests were used and to deal with the problem of multiple comparisons, attention was paid to a probability less than 0.01, although lesser significances are reported.

## Results

Data for the five types of sweet drink are found in supplementary information but, for brevity and clarity, the focus of this paper is on early exposure to cola, apple juice or fruit squash, as the other two drinks had little influence (see Supplementary Material). The choice of apple juice reflected that it was the predominant juice offered to children in the 1990s.

Table 1 reports associations between sweet drinks and adult adiposity. In males, adiposity was greater only after drinking cola (Table 1), with the exception that not drinking apple juice was associated with a higher BMI. In contrast, with females, greater adiposity was associated with drinking fruit squash and not drinking 100% fruit juice.

**Table 1:** The association between drinks consumed before 24 months and adiposity at 24 years of age.

		Males			Females		
		Cola	Apple juice	Fruit squash	Cola	Apple juice	Fruit squash
Total fat	Yes	21.9 (10.4) 535	20.1 (9.4) 666	20.5 (9.7) 1007	25.6 (11.3) 878	23.7 (10.3) 967	25.3 (11.1) 1573
MASS	No	19.5 (9.1) 718	21.0 (10.1) 582	20.5 (10.1) 241	24.2 (10.4) 1107	25.9 (11.2) 1016	22.9 (9.2) 413
(Kilograms)		*p* < 0.001	n.s.	n.s.	n.s.	*p* < 0.001	*p* < 0.005
Android fat	Yes	1.8 (1.3) 535	1.6 (1.1) 666	1.6 (1.2) 1007	1.8 (1.2) 878	1.6 (1.1) 967	1.8 (1.2) 1573
MASS	No	1.5 (1.1) 718	1.7 (1.3) 582	1.6 (1.2) 241	1.7 (1.1) 1107	1.8 (1.2) 1016	1.5 (1.0) 413
(Kilograms)		*p* < 0.001	n.s.	n.s.	n.s.	*p* < 0.001	*p* < 0.005
Body mass	Yes	25.4 (4.5) 548	24.5 (4.1) 685	24.8 (4.3) 1031	25.3 (5.7) 909	24.3 (5.1) 911	25.1 (5.6) 1622
Index	No	24.3 (4.2) 736	25.2 (4.6) 594	24.7 (4.5) 247	24.4 (5.0) 1134	25.3 (5.6) 1052	23.8 (4.5) 442
		*p* < 0.001	*p* < 0.007	n.s.	*p* < 0.06	*p* < 0.001	*p* < 0.002
Waist	Yes	874 (120) 546	855 (110) 684	861 (117) 1029	793 (130) 903	769 (115) 992	788 (126) 1619
Circumference	No	850 (113) 736	867 (124) 593	858 (118) 247	773 (115) 1135	793 (127) 1046	760 (105) 420
(Millimetres)		*p* < 0.01	n.s.	n.s.	n.s.	*p* < 0.001	*p* < 0.002

The influence of the five types of drink mentioned in the methods were used as categorical variables in a regression equation. Those who did or did not consume the drinks before two years of age (Yes/No) were compared. In no instance did the drinking of other fruit juice reach significance, although with females to a small extent having drunk other fizzy drinks was associated with greater adiposity (see supplementary information). To illustrate these effects the data are from left to right: means, standard deviations in brackets, and sample size

A question is whether these associations were related exclusively to sweet drinks, rather than a dietary pattern. At three years of age, cola, fruit squash (Table 2) or fizzy drinks (Supplementary Tables S3 and S4) were associated with consuming more energy, carbohydrate, fat, protein, and non-milk extrinsic (NME) sugars, but less non-starch polysaccharides (NSP). In contrast, apple juice was associated with a lower intake of fat and NME sugars but more protein and NSP.

**Table 2:** The association between drinks between 15 and 24 months and macro-nutrient intake at 3 years of age.

		Males			Females		
		Cola	Apple juice	Fruit squash	Cola	Apple juice	Fruit squash
Energy	Yes	5798 (1388) 2337	5249 (1289) 2061	5321 (1324) 3744	5352 (1560) 2125	5207 (1247) 1884	5239 (1309) 3492
Joules	No	5137 (1258) 2302	5288 (1359) 2558	5063 (1337) 875	5053 (1236) 2225	5193 (1349) 2459	5028 (1200) 861
		*p* < 0.001	n.s.	*p* < 0.001	*p* < 0.001	n.s.	*p* < 0.001
Carbohydrate	Yes	171.4 (47.1) 2337	166.6 (43.2) 2061	168.3 (44.5) 3744	168.9 (45.2) 2125	164.6 (41.4) 1884	165.1 (43.4) 3492
grams	No	162.2 (41.7) 2303	167.0 (45.8) 2558	160.7 (45.2) 875	158.7 (41.1) 2225	163.1 (44.8) 2459	157.8 (42.4) 861
		*p* < 0.001	n.s.	*p* < 0.001	*p* < 0.001	n.s.	*p* < 0.001
Protein	Yes	44.6 (11.5) 2337	44.8 (10.8) 2061	44.4 (11.1) 3744	45.1 (11.6) 2125	45.3 (11.0) 1884	44.7 (11.3) 3492
grams	No	43.8 (10.9) 2303	43.8 (11.4) 2558	43.4 (11.2) 875	44.1 (11.1) 2225	44.0 (11.6) 2459	44.0 (11.5) 861
		*p* < 0.02	*p* < 0.002	*p* < 0.01	*p* < 0.003	*p* < 0.001	n.s.
Fat	Yes	51.4 (14.4) 2337	49.3 (14.2) 2061	50.6 (13.9) 3744	50.9 (14.4) 2125	48.7 (13.2) 1884	49.7 (13.8) 3492
grams	No	48.5 (13.4) 2303	50.6 (14.1) 2558	47.4 (13.9) 875	47.7 (11.1) 2225	49.6 (14.2) 2336	47.4 (13.4) 861
		*p* < 0.001	*p* < 0.001	*p* < 0.001	*p* < 0.003	*p* < 0.03	*p* < 0.001
NME Sugar	Yes	52.4 (23.0) 2337	47.6 (19.4) 2061	49.8 (21.2) 3744	51.6 (21.7) 2125	47.1 (18.7) 1884	48.7 (20.4) 3492
grams	No	45.4 (18.4) 2303	50.0 (22.3) 2558	45.5 (20.7) 875	44.3 (17.8) 2225	48.5 (21.1) 2336	44.3 (18.3) 861
		*p* < 0.001	*p* < 0.001	*p* < 0.001	*p* < 0.001	*p* < 0.03	*p* < 0.001
NSP	Yes	8.7 (2.8) 2337	9.2 (3.0) 2061	8.8 (2.8) 3744	8.6 (2.8) 2125	9.1 (2.9) 1884	8.7 (2.8) 3492
grams	No	9.0 (2.9) 2303	8.6 (2.7) 2558	8.9 (3.1) 875	8.9 (3.0) 2225	8.4 (2.8) 2336	8.9 (3.0) 861
		*p* < 0.002	*p* < 0.001	n.s.	*p* < 0.001	*p* < 0.001	*p* < 0.01

The data are left to right: means, standard deviations in brackets, and sample size. Means were compared using T tests.

*NME sugar* Non-milk extrinsic sugar, *NSP* Non-starch polysaccharides.

As SSB contain neither protein nor fat, the rest of the diet must differ. Giving boys cola, fruit-squash (Table 3) or fizzy drinks (Supplementary Table S5), was associated with eating more burgers and sausages, pizza, French fries, meat, chocolate, and sweets, but less fruit. In contrast, drinking apple juice was associated with lower intakes of burgers and sausages, chocolate, sweets, puddings, or biscuits, but a higher intake of fish, fruit, green vegetables, and salad. The pattern with girls was similar.

**Table 3:** Foods associated at three years with the choice of drink when 15–24 months old.

Drinks offered 15–24 months	Cola	Apple juice	Fruit Squash
Boys diet at Three years	**More likely to eat:** Burger/sausages; Pizza; French fries; Fried food; Meat; Chocolate; Sweets. **Less likely to eat:** Fresh fruit	**More likely to eat:** Fresh fruit; Fish; Green vegetables; Salad; Pudding. **Less likely to eat:** Burger/sausage; Biscuits foods; Chocolate; Sweets.	**More likely to eat:** Burger /sausage; Pizza; French fries; Meat; Chocolate; Sweets; Cakes; Biscuits **Less likely to eat:** Fresh fruit; Fish.
Girls diet at Three years	**More likely to eat:** Burger/sausages; Pizza; French fries; Fried food; Meat; Chocolate; Sweets; Puddings; Cakes **Less likely to eat:** Fish; Fresh fruit	**More likely to eat:** Fresh fruit; Fish; Pudding; Salad **Less likely to eat:** Burger/sausage; French fries; Fried food; Meat; Chocolate; Sweets.	**More likely to eat:** Burger/sausages; Pizza; French fries; Fried food; Meat; Biscuits; Sweets; Puddings; Cakes. **Less likely to eat:** Fresh fruit; Fish; Salad

Whether children did or did not drink a particular drink at 15–24 months, was related using T tests, to the diet when aged three, reflecting a tendency to eat more or less of a food item.

Supplementary material reports the association between the drinks consumed prior to two years of age, and later diet (Supplementary Table S6). In boys, cola consumption before two years was associated with a higher energy intake between four and nine years of age. After apple juice, girls when four years of age, consumed less energy.

### Food choice and drinks

To consider the influence of the overall diet on adult adiposity, a first model of a hierarchical regression was developed based on the fourteen food items listed in the methods section. Then a second model was created by adding early exposure to sweet drinks.

In males, diet predicted body fat when 24 years (adjusted R^2^ = 0.026, F (19, 1109) = 2.598, *p* < 0.001), with French fries, burgers/sausages and root vegetables when three years, having a significant influence (Table 4). All five drinks were removed from the equation because of collinearity.

**Table 4:** Hierarchical regression equation examining relative influence of diet when three year old and the drink when young.

The significant variables were:		
	Beta	
With males the second model was significant (F(19,1109) = 2.598, *p* < 0.001). R^**2**^ = 0.026.		
French fries	0.086	*p* < 0.007
Root vegetables	0.094	*p* < 0.002
Burger/sausages	0.064	*p* < 0.039
With females the second model was significant (F (19,1749) = 2.789, *p* < 0.001; R^**2**^ = 0.020.		
Fresh fruit	−0.056	*p* < 0.026
Biscuits	−0.070	*p* < 0.005
French fries	0.055	*p* < 0.030
Burgers/sausages	0.059	*p* < 0.017

The first model included Z scores for the intake of : Burger and sausages; French fries; Fried food; Chocolate; Sweets; Biscuits; Cakes; Puddings; Fresh fruit; Green vegetables; Root vegetables; Salad; Fish; Meat; Pizza, to which were added the five drinks prior to two years to make up the second model, with total fat mass at 24 years as the dependent variable.

With females the equation was also significant (adjusted R^2^ = 0.020) F (19,1749) = 2.789, *p* < 0.001), with eating burgers/sausages and French fries being associated with greater adiposity, although those who did not eat biscuits and fresh fruit had a greater fat mass (Table 4). Not eating biscuits was possibly a reaction to a greater level of adiposity.

### Demographic variables

**Fig. 1:**
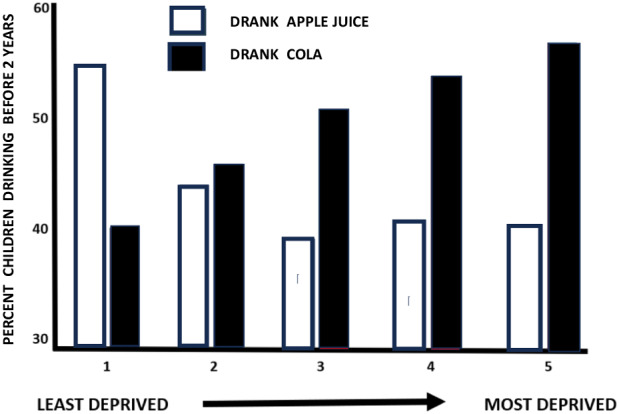
The association between deprivation and choice of drink in early life. The population was divided into quintiles based on the Multiple Index of Deprivation. The percentage of children in each of these five categories, that were given either cola or apple juice, are reported. The numbers given these drinks differed depending on the degree of deprivation (Chi Square *p* < 0.001).

Although energy consumption is often related to long-term body weight, it is possible that dietary patterns are markers for demographic variables that independently predispose to adiposity. The percentage of parents giving either cola or apple juice changed as the geographical area where the family lived became progressively more deprived (Fig. 1; Chi squared *p* < 0.001). The greater was social deprivation, the more likely it was that cola had been drunk, whereas it was less likely that apple juice had been given to child.

Supplementary material (Supplementary Table S7) lists variables associated with the provision of drinks. Cola was more likely to be given if parents were less educated, had a lower status job, the mother was younger, or either parent had a higher body weight. The early provision of apple juice was associated with an opposite pattern. These variables were included in the first model of a hierarchical regression with the five drinks subsequently then added as a second modle. Table 5 reports the significant influences in the second model. In males the BMI of the partner, the pre-birth weight of the mother and deprivation, positively influenced adiposity at 24 years. Although drinking apple juice was associated with lower adiposity, all five drinks were removed from the equation because of collinearity. Similarly, both the BMI of the partner and weight of the mother predicted the adiposity of females, but again all five drinks were removed from the equation for reasons of collinearity.

**Table 5:** Hierarchical regression equation examining relative influence of demographic variables and drink when young.

The significant variables were:		
	Beta	
With males the second model was significant (F(12,577) = 7.385, *p* < 0.001). R^**2**^ = 0.115.		
Mothers weight before pregnancy	0.240	*p* < 0.001
Partners BMI	0.133	*p* < 0.001
Deprivation	0.139	*p* < 0.001
With females the second model was significant (F (12,876) = 15.517, *p* < 0.001; R^**2**^ = 0.184.		
Mothers weight before pregnancy	0.240	*p* < 0.001
Partners BMI	0.133	*p* < 0.001

The first model included: mother’s weight before pregnancy; partners BMI; mothers social class based on occupation; partners social class based on occupation; partners highest education qualification; index of multiple deprivation; age of .mother at birth. To which were added the five drinks prior to two years to make up the second model, with total fat mass at 24 years as the dependent variable.

## Discussion

The main finding of this study was that the sweet drink given to children before two years of age was associated with adiposity at 24 years of age (Table 1). To our knowledge, this is the longest period over which such an association has been reported. There was, however, evidence that the consequences of the choice of drink relied on more than the drink itself.

In males, early exposure to cola, fruit squash (Table 2), or fizzy drinks (Supplementary Table S3), were at three years associated with a diet containing more energy, carbohydrates, fat, protein, and NME sugars, but less NSP. In contrast, early exposure to apple juice was not associated with energy intake at age three years, but there was a higher consumption of protein and NSP, and a lower consumption of fat and NME sugars. The pattern in females was similar. The choice of drink was only a part of a wider dietary pattern. Thus sugar in drinks should not be considered in isolation, as they may be associated with a healthy or less healthy diet (Tables 2 and 3). Another consideration is that FJ contains significant levels of vitamin C, folate, potassium, carotenoids, and flavonoids which are health promoting [19, 20].

The finding that dietary patterns are important was consistent with Northstone and Emmett [13], who found when diets at three, four, seven and nine years of age were compared, dietary patterns persisted over time. A ‘processed’ diet included the eating of chocolate, sweets and crisps and high fat, high sugar processed foods, but also the drinking of fizzy drinks. In contrast, fruit, vegetables, salads, pasta. and rice were part of a ‘health conscious’ diet that included fruit juice. Similarly in the present study, cola, other fizzy drinks, and fruit squash were associated with consuming burgers/sausages. pizza, fried foods, French fries, meat, chocolate, and sweets (Table 3, Supplementary Table S5). The pattern with apple juice was broadly opposite to SSB; it was less likely that burgers/sausages, fried food, and French fries were eaten (Table 3), although more fish, vegetables, and fruit were consumed.

These findings question an approach that concentrates on sugar per se, rather than a diet that happens to contain sources of sugar. Cola and apple juice both provide about 9–10 g of sugar per 100 ml, but in males cola was associated with greater adult adiposity, whereas apple juice was not (Table 1). In females, cola was also associated with higher adiposity, whereas apple juice was associated with lower adiposity (Table 1).

Such observations should not be a surprise as SSB consumption has been associated with greater adiposity when FJ consumption was not [5]. When FJ has been associated with higher body weight, 100% FJ has not been distinguished from exogenously sweetened juice [6]. The importance of this observation is illustrated by the present study, when fruit squash was found to be similar to SSB rather than FJ, in terms of macronutrient intake at three years (Supplementary Tables S4 and S6); dietary patterns at three years (Supplementary Table S5); mother’s weight and educational level; mother’s age at the child’s birth; partners BMI (Supplementary Table S7) and in females the BMI and total fat mass (Supplementary Table S2).

A reason to study early nutrition is that body fat increases after birth, and then declines only to subsequently increase from three to seven years. An earlier adiposity rebound has been associated with a greater risk of adult obesity [21, 22], such that if it occurs about three years of age rather than later, there is a greater risk of adult obesity [23], a phenomenon that occurs more often in girls [24, 25]. The question arises as whether the present findings were influenced by the age the drinks were given, as metabolic programming at critical stages in development predisposes to obesity throughout life [26]. If so, an insight into the influence of early feeding may prevent later weight gain, but attention needs to be directed to feeding in the first years of life, rather than the later ages that have often been studied in nutritional interventions.

However, the influence of diet needs to be placed in context, as although with a large sample size statistical significance was obtained, the regression equations accounted for a small amount of variance. In contrast, demographic variables had a far greater influence than the choice of drink given to the child (Table 5). Similarly, it has been reported that a child’s pattern of dietary intake is strongly influenced by socioeconomic characteristics. Factors such as a younger maternal age, and the early introduction of complementary feeding, are associated with an unhealthy dietary style [27]. Although nutritionists naturally emphasize diet, it should be assumed that a correlation between diet and adiposity reflects more than the calories provided, as the effects of demographic variables associated with diet (Table 5) had a greater influence than diet per se. A consideration of demographic variables will indicate to whom nutritional attention should be directed, and when.

A positive is that the findings add to the evidence that diet during early childhood is associated with risk of obesity in later life. As such, the attention of nutritionists should be directed to the early diet of young children, and the impact of a nutritional intervention at a younger rather than later age. There needs to be a greater emphasis on controlling energy intake in infancy and young childhood.

### Supplementary information

Table S1

Table S2

Table S3

Table S4

Table S5

Table S6

Table S7

## Data Availability

ALSPAC is a longitudinal birth cohort study that began in 1991 and continues to this day. The study encourages the use of its data and will provide access to all ‘bona fide’ researchers. Those interested should approach ALSPAC at the University of Bristol, England. Their access policy is described on their website (ALSPAC_Access_Policy.pdf (bristol.ac.uk)).
