# Supplementary material for: Early exposure to sugar sweetened beverages or fruit juice differentially influences adult adiposity
Source: Eur J Clin Nutr. 2024 Mar 15;78(6):521–6. doi: 10.1038/s41430-024-01430-y (PMC11182744; doi:10.1038/s41430-024-01430-y)
Supplement: Supplementary file 1 — Table S1 [file 41430_2024_1430_MOESM1_ESM.docx]

|  | **DRINK**  **YES/NO** | **COLA** | **FIZZY**  **DRINKS** | **APPLE JUICE** | **OTHER JUICES** | **FRUIT BASED**  **DRINKS** |
| --- | --- | --- | --- | --- | --- | --- |
| **TOTAL BONE**  **MASS**  **(Kilograms)** | **Yes**  **No** | 3.10 (0.40) 535  3.03 (0.41) 718  p< 0.003 | 3.08 (0.41) 568  3.05 (0.40) 679  n.s. | 3.07 (0.42) 666  3.06 (0.39) 582  n.s. | 3.07 (0.41) 912  3.05 (0.39) 330  n.s. | 3.06 (0.41) 1007  3.07 (0.41) 241  n.s. |
| **TOTAL LEAN**  **MASS**  **(Kilograms)** | **Yes**  **No** | 57.7 (7.1) 535  56.2 (7.3) 718  p<0.001 | 57.4 (7.2) 568  56.5 (7.4) 679  p<0.02 | 56.8 (7.1) 666  57.1 (7.5) 582  n.s. | 57.0 (7.3) 912  56.7 (7.2) 330  n.s. | 56.9 (7.2) 1007  57.0 (7.7) 241  n.s. |
| **TOTAL FAT**  **MASS**  **(Kilograms)** | **Yes**  **No** | 21.9 (10.4) 535  19.5 ( 9.1) 718  p<0.001 | 21.2 (10.0) 568  20.0 (9.5) 679  p< 0.02 | 20.1 (9.4) 666  21.0 (10.1) 582  n.s. | 20.5 (9.8) 912  20.4 (9.4) 330  n.s. | 20.5 (9.7) 1007  20.5 (10.1) 241  n.s. |
| **ANDROID FAT**  **MASS**  **(Kilograms)** | **Yes**  **No** | 1.8 (1.3) 535  1.5 (1.1) 718  p<0.001 | 1.7 (1.2) 568  1.6 (1.1) 679  p<0.02 | 1.6 (1.1) 666  1.7 (1.3) 582  n.s | 1.6 (1.2) 912  1.6 (1.2) 330  n.s. | 1.6 (1.2) 1007  1.6 (1.2) 241  n.s. |
| **BODY MASS**  **INDEX** | **Yes**  **No** | 25.4 (4.5) 548  24.3 (4.2) 736  p<0.001 | 25.2 (4.5) 584  24.4 (4.2) 694  p<0.002 | 24.5 (4.1) 685  25.2 (4.6) 594  p<0.005 | 24.8 (4.2) 933  24.6 (4.4) 338  n,s. | 24.8 (4.3) 1031  24.7 (4.5) 247  n.s. |
| **WAIST**  **CIRCUMFERENCE**  **(Millimetres)** | **Yes**  **No** | 874 (120) 546  850 (113 736  p<0.001 | 870 (121) 582  853 (112) 694  p<0.006 | 855 (110) 684  867 (124) 593  n.s. | 860 (117) 931  859 (116) 338  n.s. | 861 (117) 1029  858 (118) 247  n.s. |

**Table S1 The association between drinks consumed by boys before 24 months and adiposity at 24 years of age.** The data are from left to right: means, standard deviations in brackets and sample size
