# Supplementary material for: Early exposure to sugar sweetened beverages or fruit juice differentially influences adult adiposity
Source: Eur J Clin Nutr. 2024 Mar 15;78(6):521–6. doi: 10.1038/s41430-024-01430-y (PMC11182744; doi:10.1038/s41430-024-01430-y)
Supplement: Supplementary file 2 — Table S2 [file 41430_2024_1430_MOESM2_ESM.docx]

|  | **DRINK**  **YES/NO** | **COLA** | **FIZZY**  **DRINKS** | **APPLE JUICE** | **OTHER JUICES** | **FRUIT BASED**  **DRINKS** |
| --- | --- | --- | --- | --- | --- | --- |
| **TOTAL BONE**  **MASS**  **(Kilograms)** | **Yes**  **No** | 2.35 (0.29) 878  2.35 (0.29) 1107  n.s. | 2.37 (0.28 ) 943  2.34 (0.20) 1040  p<0.01 | 2.36 (0.28) 967  2.35 (0.29) 1016  n.s. | 2.35 (0.29) 1431 2.35 (0.28) 533  n.s. | 2.36 (0.29) 1573  2.33 (0.28) 413  n.s. |
| **TOTAL LEAN**  **MASS**  **(Kilograms)** | **Yes**  **No** | 41.2 (5.5) 878  41.1 (5.4) 1107  n.s. | 41.5 ( 5.4) 943  40.8 (5.3) 1040  p<0.006 | 41.3 (5.3) 967  41.1 (5.5) 1016  n.s. | 41.1 (5.4) 1431  41.2 (5.4) 533  n.s. | 41.2 (5.5 ) 1573  40.9 (5.2 ) 413  n.s. |
| **TOTAL FAT**  **MASS**  **(Kilograms)** | **Yes**  **No** | 25.6 (11.3) 878  24.2 (10.4) 1107  p<0.004 | 25.8 (11.7) 943  23.9 (9.8) 1040  p<0.001 | 23.7 (10.3) 967  25.9 (11.2) 1016  p<0.001 | 24.6 (10.6) 1431  25.2 (11.1) 533  n.s. | 25.3 (11.1 ) 1573  22.9 ( 9.2 ) 413  p<0.001 |
| **ANDROID FAT**  **MASS**  **(Kilograms)** | **Yes**  **No** | 1.8 ( 1.2) 878  1.7 (1.1) 1107  p<0.006 | 1.8 (1.3) 943  1.6 (1.1) 1040  p<0.001 | 1.6 (1.1) 967  1.8 (1.2) 1016  p<0.001 | 1.7 (1.2 ) 1431  1.8 (1.2 ) 533  n.s. | 1.8 (1.2 ) 1573  1.5 (1.0 ) 413  p<0.001 |
| **BODY MASS**  **INDEX** | **Yes**  **No** | 25.3 (5.7) 909  24.4 (5.0) 1134  p<0.001 | 25.3 (5.8) 972  24.3 (4.9) 1071  p<0.001 | 24.3 (5.1) 991  25.3 (5.6) 1052  p<0.001 | 24.7 (5.3) 1470  25.0 (5.6) 551  n.s. | 25.1 (5.6 ) 1622  23.8 (4.5 ) 442  p <0.001 |
| **WAIST**  **CIRCUMFERENCE**  **(Millimetre)** | **Yes**  **No** | 793 (130) 903  773 (115) 1135  p<0.001 | 794 (131) 970  771 (111) 1068  p<0.001 | 769 (115) 992  795 (127) 1046  p<0.001 | 779 (120) 1467  787 (126) 550  n.s. | 788 (126) 1619  760 (105) 420  p<0.001 |

**Table S2 The association between drinks consumed by girls before 24 months and adiposity at 24 years of age** The data are from left to right: means, standard deviations in brackets and sample size. Differences assessed using T test.
