# Supplementary material for: Early exposure to sugar sweetened beverages or fruit juice differentially influences adult adiposity
Source: Eur J Clin Nutr. 2024 Mar 15;78(6):521–6. doi: 10.1038/s41430-024-01430-y (PMC11182744; doi:10.1038/s41430-024-01430-y)
Supplement: Supplementary file 3 — Table S3 [file 41430_2024_1430_MOESM3_ESM.docx]

|  |  | **COLA** | **OTHER FIZZY**  **DRINKS** | **APPLE JUICE** | **OTHER JUICES** | **FRUIT-BASED**  **SQUASH** |
| --- | --- | --- | --- | --- | --- | --- |
| **ENERGY**  **(KILOJOULES)** | **Yes No** | 5.40 (1.39) 2337  5.14 (1.26) 2303  p<0.001 | 5.40 (1.37) 2339  5.14 (1.28) 2288  p<0.001 | 5.25 (1.29) 2061  5.29 (1.36) 2558  n.s. | 5.31 (1.32) 3252  5.18 (1.32) 1335  p<0.001 | - 5.32 (1.32) 3744  5.06 (1.34) 875  p<0.001 |
| **CARBOHYDRATE**  **(GRAMS)** | **Yes No** | 171.4 (47.1) 2337  162.2 (41.7) 2303  p<0.001 | 171.2 (48.2) 2339  162.2 (42.7) 2288  p<0.001 | 166.6 (43.2) 2061  167.0 (45.8) 2558  n.s. | 168.1 (44.4) 3252  163.8 (45.1) 1335  p<0.003 | 168.3 (44.5) 3744  160.7 (45.2) 875  p<0.001 |
| **PROTEIN**  **(GRAMS)** | **Yes No** | 44.6 (11.5) 2337  43.8 (10.9) 2303  p<0.02 | 44.6 (11.3) 2339  43.8 (11.1) 2288  p<0.01 | 44.8 (10.8} 2061  43.8 (11.4) 2558  p<0.002 | 44.8 (11.1) 3252  42.7 (11.1) 1335  p<0.001 | 44.4 (11.1) 3744  43.4 (11.2) 875  p<0.01 |
| **FAT**  **(GRAMS)** | **Yes No** | 51.4 (14.4) 2337  48.5 (13.4) 2303  p<0.001 | 51.3 (14.4) 2339  48.7 (13.4) 2288  p<0.001 | 49.3 (14.2) 2061  50.6 (14.1) 2558  p<0.001 | 50.3 (13.9) 3252  49.4 (13.9) 1335  p<0.05 | 50.6 (13.9) 3744  47.4 (13.9) 875  p<0.001 |
| **NME sugars**  **(GRAMS)** | **Yes No** | 52.4 (23.0) 2337  45.4 (18.4) 2303  p<0.001 | 52.4 (22.8) 2339  45.4 (18.6) 2288  p<0.001 | 47.6 (19.4) 2061  50.0 (22.3) 2558  p<0.001 | 49.3 (20.8) 3252  48.0 (21.6) 1335  n.s. | 49.8 (21.2) 3744  45.4 (20.7) 875  p<0.001 |
| **NSP**  **(GRAMS)** | **Yes No** | 8.7 (2.8) 2337  9.0 (2.9) 2303  p<0.002 | 8.7 (2.7) 2339  9.0 (3.0) 2288  p<0.006 | 9.2 (3.0) 2961  8.6 (2.7) 2558  p<0.0 01 | 9.0 ( 2.9) 3252  8.6 (2.8) 1335  p<0.001 | 8.8 (2.8) 3744  8.9 (3.1) 875  n.s. |

**Table S3 The influence in boys of drinks between 15 and 24 months and macro-nutrient intake at 3 years of age.**

The data are left to right: means, standard deviations in brackets, and sample size. Differences assessed with T tests
