# Supplementary material for: Early exposure to sugar sweetened beverages or fruit juice differentially influences adult adiposity
Source: Eur J Clin Nutr. 2024 Mar 15;78(6):521–6. doi: 10.1038/s41430-024-01430-y (PMC11182744; doi:10.1038/s41430-024-01430-y)
Supplement: Supplementary file 4 — Table S4 [file 41430_2024_1430_MOESM4_ESM.docx]

|  |  | **COLA** | **OTHER FIZZY**  **DRINKS** | **APPLE JUICE** | **OTHER FRUIT**  **JUICES** | **FRUIT-BASED**  **SQUASH** |
| --- | --- | --- | --- | --- | --- | --- |
| **ENERGY**  **(KILOJOULES)** | **Yes**  **No** | 5.35 (1.36) 2125  5.05 (1.24) 2225  p<0.001 | 5.34 (1.34) 2209  5.05 (1.26) 2136  p<0.001 | 5.21 (1.25) 1884  5.19 (1.35) 2459  n.s. | 5.26 (1.30) 3102  5.07 (1.33) 1205  p<0.001 | 5.24 (1.31) 3492  5.03 (1.21) 861  p<0.001 |
| **CAROHYDRATE**  **(GRAMS)** | **Yes No** | 168.9 (45.2) 2125  158.7 (41.0) 2225  p<0.001 | 168.5 (44.3) 2209  158.7 (41.9) 2136  p<0.001 | 164.6 (41.4) 1884  163.1 (44.8) 2136  n.s. | 165.8 (43.1) 3102  158.5 (43.9) 1205  p<0.001 | 165.1 (43.4) 3492  157.8 (42.4 ) 861  p<0.001 |
| **PROTEIN**  **(GRAMS)** | **Yes No** | 45.1 (11.6) 2125  44.1 (11.1) 2225  p<0.003 | 45.0 (11.5) 2209  44.1 (11.2) 2136  p<0.01 | 45.3 (11.0) 1884  44.0 (11.6 ) 2459  p<0.001 | 45.1 (11.3) 3102  43.1 (11.5) 1205  p<0.001 | 44.7 (11.3) 3492  44.0 ( 11.5) 861  n.s. |
| **FAT**  **(GRAMS)** | **Yes No** | 50.9 (14.4) 2125  47.7 (12.9) 2225  p<0.001 | 50.7 (14.2) 2209  47.7 (13.1) 2136  p<0.001 | 48.7 (13.2) 1884  49.6 (14.2) 2336  p<0.03 | 49.6 (13.7) 3102  48.5 (14.0) 1205  p<0.03 | 49.7 (13.8) 3492  47.4 (13.4) 861  p<0.001 |
| **NME SUGARS**  **(GRAMS)** | **Yes No** | 51.6 (21.7) 2125  44.3 (17.8) 2225  p<0.001 | 51.1 (21.1) 2209  44.4 (18.5) 2136  p<0.001 | 47.1 (18.7) 1884  48.5 (21.1) 2336  p<0.03 | 48.7 (20.1) 3102  45.9 (20.1) 1205  p<0.001 | 48.7 (20.4) 3492  44.3 (18.3) 861  p<0.001 |
| **NSP**  **(GRAMS)** | **Yes No** | 8.6 (2.8) 2125  8.9 (2.9) 2225  p<0.001 | 8.6 (2.8) 2209  8.8 (2.9) 2136  p<0.05 | 9.1 (2.9) 1884  8.4 (2.8 ) 2336  p<0.001 | 8.9 (2.8) 3102  8.3 (2.8) 1205  p<0.001 | 8.7 (2.8) 3492  8.9 (3.0) 861  p<0.01 |

**Table S4 The influence in girls of drinks between 15 and 24 months on macro-nutrient intake at 3 years of age T**

The data are left to right: means, standard deviations in brackets, and sample size. Differences assessed with T tests.
