# Supplementary material for: Early exposure to sugar sweetened beverages or fruit juice differentially influences adult adiposity
Source: Eur J Clin Nutr. 2024 Mar 15;78(6):521–6. doi: 10.1038/s41430-024-01430-y (PMC11182744; doi:10.1038/s41430-024-01430-y)
Supplement: Supplementary file 5 — Table S5 [file 41430_2024_1430_MOESM5_ESM.docx]

| **DRINKS OFFERED**  **15-24 MONTHS** | **COLA** | **OTHER FIZZY**  **DRINKS** | **APPLE JUICE** | **OTHER FRUIT**  **JUICE** | **SQUASH** |
| --- | --- | --- | --- | --- | --- |
| **BOYS DIET AT**  **THREE YEARS** | **More likely to eat:**  Burger/sausages; Pizza; French fries; Fried food; Meat; Chocolate; Sweets.  **Less likely to eat:**  Fresh fruit | **More likely to eat**:  Burger/sausages; Pizza; Fried food; French fries: Meat; Chocolate; Sweets.  **Less likely to eat:**  Fresh fruit | **More likely to eat:**  Fresh fruit; Fish;  Green vegetables; Salad; Pudding.  **Less likely to eat:**  Burger/sausage; Biscuits foods; Chocolate; Sweets. | **More likely to eat:**  Fresh fruit; Fish, Cakes; Pudding’ Salad.  **Less likely to eat:** | **More likely to eat:**  Burger /sausage;  Pizza; French fries; Meat; Chocolate; Sweets; Cakes; Biscuits  **Less likely to eat:**  Fresh fruit; Fish. |
| **GIRLS DIET AT**  **THREE YEARS** | **More likely to eat:**  Burger/sausages; Pizza; French fries; Fried food; Meat; Chocolate; Sweets; Puddings; Cakes  **Less likely to eat:**  Fish; Fresh fruit | **More likely to eat:**  Burger/sausages; Pizza; French fries; Fried food; Meat; Chocolate; Sweets; Puddings; Cakes  **Less likely to eat:**  Fish; Fresh fruit. | **More likely to eat:**  Fresh fruit; Fish; Pudding; Salad  **Less likely to eat:**  Burger/sausage; French fries; Fried food; Meat; Chocolate; Sweets. | **More likely to eat:**  Fresh food; Fish; Pudding; Green vegetables; Root vegetables  **Less likely to eat:**  Burgers/sausage; French fries; Meat; Cake | **More likely to eat:**  Burger/sausages; Pizza; French fries; Fried food; Meat; Biscuits; Sweets; Puddings; Cakes.  **Less likely to eat:**  Fresh fruit; Fish; Salad |

**TABLE S5 The diet when three years of age was associated with the choice of drinks when 15 to 24 months old.**

Whether children did or did not drink particular drinks at 15 to 24 months was related, using t tests, to the diet when aged three, measured by a food frequency questionnaire. The findings reflect whether consuming the drink was associated with a statistically significant tendency to eat more or less of a food item.
