# Supplementary material for: Early exposure to sugar sweetened beverages or fruit juice differentially influences adult adiposity
Source: Eur J Clin Nutr. 2024 Mar 15;78(6):521–6. doi: 10.1038/s41430-024-01430-y (PMC11182744; doi:10.1038/s41430-024-01430-y)
Supplement: Supplementary file 6 — Table S6 [file 41430_2024_1430_MOESM6_ESM.docx]

|  |  | **MALES** | |  | **FEMALES** | |
| --- | --- | --- | --- | --- | --- | --- |
|  |  | **COLA** | **APPLE JUICE** |  | **COLA** | **APPLE JUICE** |
| **4 Years** | **Yes**  **No** | 6095 (1467) 2243  5872 (1322) 2257  p< 0.001 | 5938 (1324) 2009  6019 (1457) 2473  p<0.05 |  | 5936 (1386) 2055  5683 (1231) 2139  p<0.001 | 5815 (1313) 1818  5796 (1322) 2361  n.s. |
| **7 Years** | **Yes**  **No** | 7897 (1790) 1913  7672 (1791) 2035  p<0.001 | 7726 (1789) 1785  7824 (1934) 2150  n.s. |  | 7715 (1817) 1811  7323 (1620) 1942  p<0.001 | 7506 (1732) 1651  7515 (1736) 2093  n.s. |
| **9 Years** | **Yes**  **No** | 8259 (2079 ) 1753  7985 (1905) 1874  p<0.001 | 8129 (1919) 1658  8103 (2056) 1957  n.s. |  | 7963 (1965) 1651  7639 (1753) 1841  p<0.001 | 7790 (1839) 1555  7793 (1881) 1928  n.s. |
| **11 Years** | **Yes**  **No** | 8141 (1688 ) 1500  8249 (1606) 1678  n.s. | 8200 (1624) 1483  8201 (1663) 1685  n.s |  | 7376 (1477) 1511  7408 (1431) 1676  n.s. | 7419 (1380) 1447  7375 (1508) 1737  n.s. |
| **13 Years** | **Yes**  **No** | 8935 (2340 ) 1204  9058 (2168 ) 1409  n.s. | 9057 (2250) 1242  8958 (2252) 1361  n.s. |  | 7341 (1856) 1217  7554 (1769) 1461  p<0.002 | 7598 (1824) 1259  7340 (1780) 1413  p<0.001 |

**Table S6 The association between the drink consumed prior to two years and energy intake up to 13 years of age.**

Those who did or did not consume the drinks before two years of age (Yes / No) were compared in terms of the total energy consumed up to 13 years of age. The data are mean joules, standard deviation in brackets and sample size.
