# Supplementary material for: Early exposure to sugar sweetened beverages or fruit juice differentially influences adult adiposity
Source: Eur J Clin Nutr. 2024 Mar 15;78(6):521–6. doi: 10.1038/s41430-024-01430-y (PMC11182744; doi:10.1038/s41430-024-01430-y)
Supplement: Supplementary file 7 — Table S7 [file 41430_2024_1430_MOESM7_ESM.docx]

| **DRINKS GIVEN TO INFANT BETWEEN 15 AND 24 MONTHS** | | | | | |
| --- | --- | --- | --- | --- | --- |
|  | **COLA** | **FIZZY DRINKS** | **APPLE JUICE** | **OTHER JUICES** | **FRUIT BASED**  **DRINKS** |
| **Mothers pre-natal weight in pounds** | Yes 138.0 (25.2)  No 133.8 (22.0)  p<0.001 | Yes 137.6 (24.9)  No 134.1 (21.9)  p<0.00 1 | Yes 134.8 (22.3)  No 136.8 (24.7)  p<0.001 | Yes 135.8 (23.7)  No 136.0 (23.7)  n.s. | Yes 136.5 (23.7)  No 133.3 (23.5)   - p<0.001 |
| **Mother social class based on occupation** | Yes 3.3 (1.2)  No 2.9 (1.2)  p<0.001 | Yes 3.2 (1.2)  No 3.0 (1.3)  p< 0.001 | Yes 2.9 (1.2)  No 3.3 (1.2)  p<0.02 | Yes 2.9 (1.2)  No 3.3 (1.2)  p<0.001 | Yes 3.1 (1.2)  No 3.2 (1.2)  n.s. |
| **Age of mother**  **at birth** | Yes 27.9 (4.6)  No 29.6 (4.5)  p<0.001 | Yes 28.3 (4.6)  No 29.2 (4.7)  p< 0.001 | Yes 29.7 (4.6)  No 28.0 (4.5)  p<0.001 | Yes 28.9 (4.7)  No 28.3 (4.7)  p<0.001 | Yes 28.6 (4.6)  No 29.6 (4.6)  p<0.001 |
| **Index of Multiple**  **Deprivation**  **(Quintiles)** | Yes 2.9 (1.3)  No 2.6 (1.3)  p<0.001 | Yes 2.88 (1.3)  No 2.58 (1.3)  p<0.001 | Yes 2.56 (1.3)  No 2.87 (1.3)  p<0.001 | Yes 2.7 ( 1.3)  No 2.8 (1.3)  p<0.01 | Yes 2.7 (1.3)  No 2.7 (1.3)  n.s. |
| **Partners BMI** | Yes24.5 (3.2)  No 24.1 (3.1)  p<0.001 | Yes 24.6 (3.3)  No 24.0 (3.0)  p<0.001 | Yes 24.2 (3.0)  No 24.5 (3.3)  p<0.003 | Yes 24.3 (3.1)  No 24.5( 3.3)  n.s. | Yes 24.4 (3.1)  No 24.1 (3.1)  p<0.04 |
| **Partners highest**  **education level** | Yes 3.1 (1.4)  No 3.6 (1.3)  p<0.001 | Yes 3.1 ( 1.3)  No 3.5 (1.3)  p<0.001 | Yes 3.7 (1.3)  No 3.0 (1.3)  p<0.001 | Yes 3.4 (1.3)  No 3.2 (1.3)  p<0.001 | Yes 3.3 (1.3)  No 3.5 (1.3)  p<0.001 |
| **Partners social class**  **based on occupation** | Yes 3.2 (1.2)  No 2.9 (1.2)  p<0.001 | Yes 3.2 (1.3)  No 2.9 (1.2)  p<0.001 | Yes 2.7 (1.2)  No 3.3 (1.2)  p<0.001 | Yes 3.0 (1.2)  No 3.2 (1.2)  p<0.001 | Yes 3.1 (1.3)  No 2.9 (1.2)  p<0.001 |

**Table S7 Parental characteristics and the choice of drink before 24 months**

The data are means and standard deviations. Where a higher score reflected a longer formal education; a lower score for social class reflected occupations with more training and responsibility; a higher score indicated greater deprivation. Responses compared using T tests.
